# Bending Fatigue Behaviors Analysis and Fatigue Life Prediction of 20Cr2Ni4 Gear Steel with Different Stress Concentrations near Non-metallic Inclusions

**DOI:** 10.3390/ma12203443

**Published:** 2019-10-21

**Authors:** Zhiguo Xing, Zhiyuan Wang, Haidou Wang, Debin Shan

**Affiliations:** 1 National Key Lab for Remanufacturing, Academy of Armored Forces Engineering, Beijing 100072, China; xingzg2011@163.com (Z.X.); reincarnational@163.com (Z.W.); 2 School of Materials Science and Engineering, Harbin Institute of Technology, Harbin 150001, China; shandb@hit.edu.cn

**Keywords:** bending fatigue behavior, non-metallic inclusions, stress concentrations, quantitative analysis

## Abstract

To investigate the relationship between inclusions and bending fatigue behaviors in 20Cr2Ni4 steel under different stress concentrations. This paper designs a new experimental method to prefabricate different size stress concentrations near the inclusions, and then conducts a new type of bending fatigue test to study the inclusions and their surrounding stress distributions in 20Cr2Ni4 steel. A microhardness tester was combined with laser etching equipment to realize the prefabrication of different stress concentrations at arbitrary positions around any inclusion on the gear steel surface. This method provides an experimental basis for the quantitative analysis of the relationship between stress distribution and fatigue life around the inclusions of heavy-duty gear steels. We also predict the bending fatigue lives of heavy-duty gear steels with different types of inclusions, stress states, and spatial distributions. Then, based on the prefabricated notch parameters and the state of inclusions in the steel, a mathematical model of quantitative analysis is proposed, which can accurately predict the fatigue limit of heavy-duty gear steel. The research results can be applied to the actual use of heavy-duty gears and to the accurate life estimation based on the state of gear stress, thereby providing a quantitative reference model for subsequent gear steel production and gear part processing.

## 1. Introduction

A wide range of industrial applications use 20Cr2Ni4 alloys because of their excellent combination of mechanical ductility and fatigue resistance [1]. This alloy has the characteristics of good hardenability, surface hardness, wear resistance, toughness (after carburizing and quenching), low-temperature impact toughness, anti-contact fatigue, and gluing ability [2]. The high strength, low density, and good formability characteristics of 20Cr2Ni4 are desirable for heavy-duty impulse system gears. In the large-scale mechanical equipment research field, there have been extensive studies regarding the implementation of 20Cr2Ni4 in the manufacturing of gears, rolling bearings, and pin bearings [3,4,5].

Currently, the international mechanical industry is trending to a large-scale and complex high-speed and high-duty service environment, especially in the fields of aerospace, armored tanks, and the automobile industry [6]. The material used in those manufacturing processes is required to have high mechanical properties, such as those of the high-strength steel 20Cr2Ni4 in this paper. Unfortunately, bending fatigue failure often occurs in 20Cr2Ni4 in practical engineering applications and experimental tests. Bending fatigue failure occurs instantaneously without any warning, and this type of failure is irreversible. Bending fatigue failures often cause major accidents, terrible economic losses, and substantial effects on mechanical systems [7]. With the further study of the bending fatigue failure behaviors of 20Cr2Ni4 materials, it has been found that many factors can cause bending fatigue failures, which has highly random properties. Moreover, each failure occurs in the three stages of crack initiation, crack source expansion, and fatigue fracture, all of which are highly correlated with the state of inclusions in steel [8].

The bending fatigue failures of heavy-duty gears studied in this paper usually occur due to surface and near-surface inclusions, and there are differences between the elastic moduli of the inclusions and that of the matrix materials. In the service process, an inclusion under the same stress as the matrix produces a different strain, forms a common point of fatigue crack nucleation, gradually expands, and eventually leads to fatigue failure [9]. Therefore, it can be seen that it is good to improve the bending fatigue property of a material system by building a system to withstand the small stress concentrations of inclusions in a material. Thus, accurately predicting the stress load distribution around inclusions has become a research hotpot, and researchers at home and abroad have provided many reliable methods. Krewerth et al. [10] processed the center part of the sample into a circular arc. According to the characteristics of the stress concentration at the vertex of the circular arc, they either analyzed the shape and hardness of the inclusions or analyzed the combination of these features with matrix state characteristics of cyclic shear stress under the influence of the fatigue crack initiation and propagation behavior. Tang et al. [11], based on the viewpoint of damage mechanics, used Abaqus software to design a 1/8 circular notched specimen. A new two-scale model was used to describe the bending fatigue damage evolution mechanism. Chen Yanqiang [12] of Dalian University of Technology used a linear cutting method to prefabricate small gaps in three-point bending samples to study the effects of strong stress concentrations on bending fatigue behaviors. This method provided a new way to study fatigue crack propagation. However, the stress concentration provided by artificial prefabricated macro defects was too large, which also caused great damage to the matrix. Moreover, this method has difficultly establishing a direct relation between the force state and the bending fatigue behavior. Roiko et al. [13] used a focused ion beam (FIB) to cut out small incision specimens and small holes and then performed different fatigue loadings on the sample loading to study the effects of stress loads on fatigue crack propagations. Although this practice helped to better simulate parts of the stresses of inclusions, FIB directly cuts small cracks into the samples that act as a primary source for crack initiation.

In recent years, with the continuous development of laser equipment, prefabricating various shapes on the surface of materials has become a popular research area [14]. Laser etching technology uses high-energy laser beams to directly melt the substrate surface from a set of mechanical structures on the matrix [15]. The laser etching method can form different defect states on the metal base surface to prefabricate different stress concentrations. Moreover, the laser etching method will not crack the sample, break samples, or parts of the organization structure; therefore, the laser etching method produces a controlled stress concentration near the inclusion, which can minimize the damage to the base material [16,17].

The purpose of this study is to predict the fatigue lives of steel inclusions and analyze the bending fatigue behaviors caused by inclusions. This study is used to semi-quantitatively describe the bending fatigue performance of steel in the presence of stress concentrations around inclusions. In inclusion and bending fatigue performance studies in gear steel, ensuring that the prefabricated defects are accurately set up around the steel inclusion is a problem. In this paper, laser etching equipment is used to locate prefabricated defects near inclusions. The mechanism of bending fatigue failure of gear steel is studied by artificial stress concentrations. The method centers the surface of the metal part, which is artificially generated with different stress concentrations, around the inclusions to study the bending fatigue failure mechanism of the gear steel. Hereafter, the areas where inclusions are apparently found on the surfaces of heavy-duty gear steels and the different loading states of bending fatigue loads are prefabricated to establish a system of loads and inclusions. Finally, according to the Murakami fatigue fracture threshold design model established by Tanaka and Akiniwa [18] according to Paris formula design of bending fatigue life, a new fatigue strength model is proposed to make semi-quantitative predictions; this new model characterizes non-metallic inclusions in the stress concentration around the influence of bending fatigue load of gear steel, thus improving targeted non-metallic inclusions for reinforcement, obtaining qualitative predictions of the overload in steel parts, and meeting the demand of various important engineering applications [19].

## 2. Materials and Methods

### 2.1. Materials and Specimens

The 20Cr2Ni4 heavy-duty gear steel used in the experiment is Cr-Ni gear steel. The steel was drawn in accordance with national standard GB/T 3077-2015, and the composition of the steel is detailed in Table 1. Under the standard, the content of harmful inclusions in gear steel is significantly reduced, and the distribution of non-metallic inclusions in the steel is uniform, which is convenient for studying the bending fatigue damage behavior of heavy-duty gear steel.

After drawing the 20Cr2Ni4 steel, the blank is processed into standard tensile specimens of bone shape for inspection. Samples were conducted in INSTRON5985 electronic universal material testing machine in accordance with GB/T 228-2002 standard, with a standard distance of 20 mm and a strain rate of 10^−2^/s to determine the basic mechanical properties of steel, including tensile strength, elastic modulus, yield strength, section elongation, section shrinkage, and hardness, as shown in statistical Table 2.

**Figure 1:**
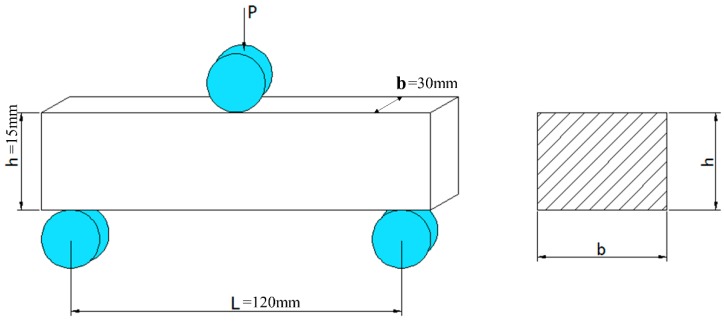
Sample size and experimental mounting diagram.

In this experiment, the steel was cut according to the specification in Figure 1. Since the fatigue property of the metal material was closely related to the surface grain orientation and roughness of the sample, the sample was processed in strict accordance with the relevant requirements of GB/T232-2010 to prepare 60 groups of samples. The samples were divided into three groups for fatigue testing, and at least 15 valid data were obtained in each group. For the samples, the height h = 15 mm, thickness b = 2 h, sample length S = 7 b, and span L > 4 h which value is tested as 60 mm in the actual test. However, if the sample size exceeds 150 mm in the actual test, the test machine and sample cannot reach resonance, and fatigue load cannot be loaded. Therefore, 120 mm is selected as the test standard for l; the other processing factors can be found in GB/T 2975. It is worth mentioning that the radius of the inverted circle is 3 mm, and the edges should not form burrs or scars that affect the test results.

### 2.2. Experimental Procedures

**Figure 2:**
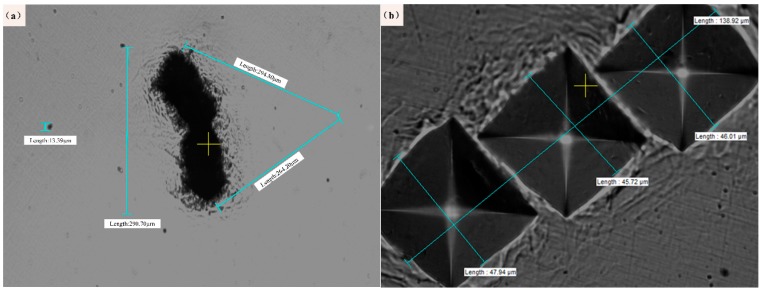
Locations for indentation and inclusions under different magnifications: (**a**) inclusions and indentation position under 20× magnification and (**b**) standard indentation under 50× magnification.

First, the presence of non-metallic inclusions on the steel surface was evaluated, and inclusion field detection equipment (Aspex Explorer automatic analyzer, Central Iron and Steel Research Institute, Beijing, China.) was used for characterization. In the test, the steel was cut into rectangular samples with thicknesses of 5 mm and lengths and widths of 10 mm, which were polished to mirror finish and then put into the Aspex Explorer automatic analyzer. This instrument combined with scanning electron microscopy (SEM) and energy-dispersive X-ray spectroscopy (EDS) can automatically search and measure inclusions in clean steel. In this paper, the instrument was used for testing, and the statistical area was 27.812 mm^2^, and the number of inclusions was 1043. Preparation for the three-point bending test is then performed according to the guidelines in Section 2.1. After the three-point bending test is performed according to the guidelines in Section 2.1, the middle area of the surface of each sample is polished. After sanding with a sequence of 500, 1000, 1200, and 2000 grit sandpaper, the inclusions can be seen in the area under a 20× magnification (as shown in Figure 2a). When looking for inclusions on the surface, the priority choice is the middle area, where the stresses concentrate and the source of crack initiation most easily occurs; in the subsequent three-point bending fatigue test, the effective span is greater than 150 mm. In the vicinity of the selected inclusion, a hardness meter is used to create multiple indentations. After several experiments, it was found that the diameter of each average indentation produced by the 500 g indenter was between 40 and 50 μm. It is easier to observe the sample with the naked eye when trying to avoid problems such as excessive gap and disordered orientation. Finally, it is concluded that this method requires at least three tags, as shown in Figure 2b (diameter 138.92 µm). This labelling method allows the trace to be visible to the naked eye to the extent that the sample is sufficiently small.

**Figure 3:**
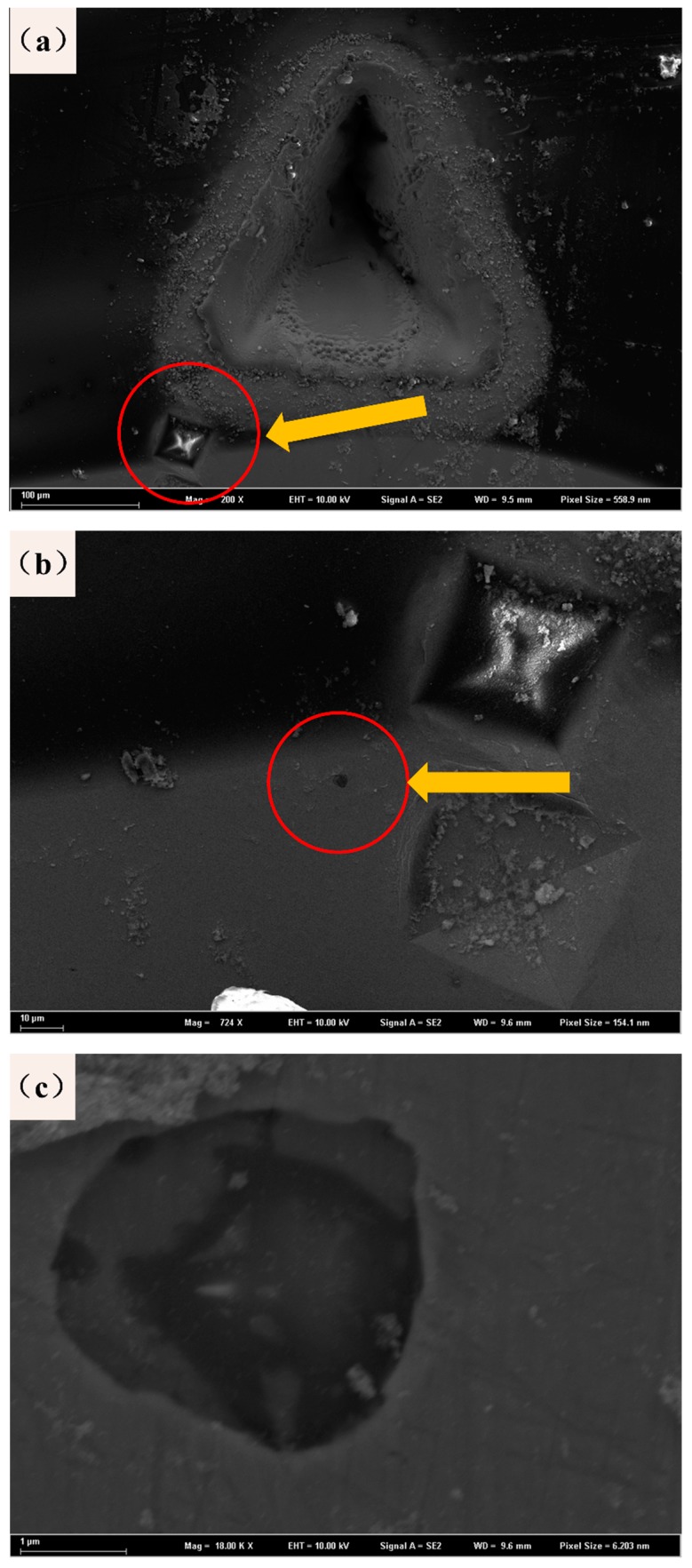
SEM images of the laser-prefabricated defects: (**a**) overall rendering, (**b**) indentation rendering, and (**c**) inclusion rendering.

Laser etching equipment was used to create triangular or circular blind holes at the mark. To clearly reflect the experimental process, a group of etched parts was selected for description. Figure 3 shows the triangular blind holes and the indentation from the microhardness tester, which is located in the red circle; this figure shows that the triangular blind hole does not cover the indentation. This offset is related to the error of the experiment and the large diameter of the blind hole. The area in the red circle is magnified to obtain the picture shown in Figure 3b, and it can be found that there are obvious black particles beside the marks. Finally, it is confirmed that an effective sample contains a blind hole covering the indentation and does not affect the inclusion state. There are 40 effective samples, each of which is divided into 20 round hole samples and 20 angular notched samples, and the external circle diameter of the circular hole and the triangular hole are both 0.2 mm.

To better simulate the actual service state of a heavy-duty gear, we need to perform the same heat treatment process with the reload gear for the prefabricated sample. The process is as follows: Normalizing, carburizing, high-temperature tempering, quenching, and low-temperature tempering. The specific temperature control is shown in Table 3.

**Figure 4:**
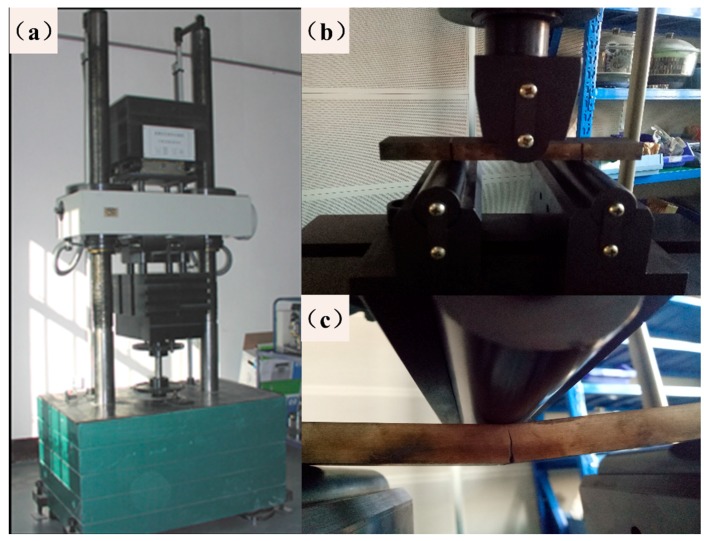
Images of the three-point bending specimen loading: (**a**) testing machine, (**b**) sample before loading, and (**c**) sample after loading.

After all the samples were heat-treated, bending fatigue tests were performed on a PLG-300C high-frequency tensile testing machine (as shown in Figure 4a). The maximum average load of this equipment is ±300 kN, and the maximum alternating load is ±150 kN. The frequency range is 80–250 Hz. As there is no definite standard for three-point bending fatigue tests, the test specimen is loaded in accordance with the national standard GB/T 232-2010 (as shown in Figure 4b). Then, the bending fatigue tests were conducted, and the specific setting parameters were as follows: The span was 120 mm, the dynamic load is 20–30 kN, the stress ratio is 0.1, and the static load follows the test machine at a frequency of 67 Hz. The test was performed until the sample was broken or until reaching 3 × 10^6^ cycles (as shown in Figure 4c), and the bending fatigue limit of the steel at 3 × 10^6^ cycles was determined by the single sample method. The Basquin model equation was used to fit the data, and the *S-N* curves of each group were obtained to predict the bending fatigue limit. 

The fracture surfaces were observed by field emission scanning electron microscopy (FESEM, ZEISS Supra55/3195: the test equipment is provided by the Institute of Automation, Chinese Academy of Sciences in Beijing, China.). When an inclusion was found at the fracture origin, the kind of inclusion was identified by EDS.

## 3. Experimental Results

### 3.1. Analysis of the Inclusions in Steel

**Figure 5:**
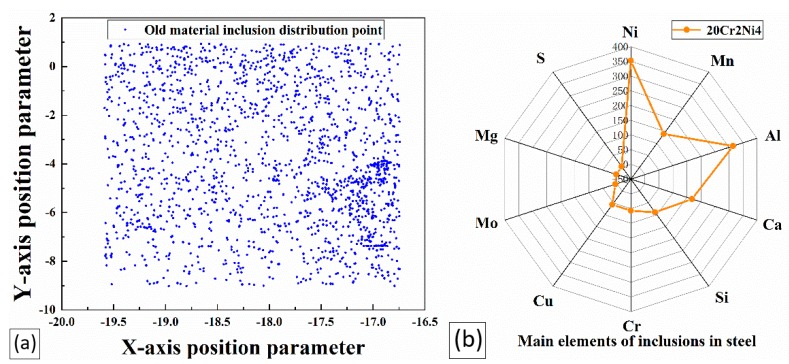
Characterization of steel inclusions: (**a**) overall distribution location map, (**b**) the number of major element inclusions in the area.

The overall distribution results of the inclusions are shown in Figure 5a. In the figure, the distribution area of the inclusions is enlarged in the same proportion to better show the distribution in the picture. The distribution status of the inclusions in the figure is directly compared, and the distribution rules are summarized. The inclusions were unevenly distributed in the steel, and some local inclusions were concentrated. Figure 5b shows a radar diagram of the distribution of inclusions according to the statistical number of the inclusion types. The highest element content in the inclusions was then defined. It can be directly seen from the figure that the largest number of inclusions in the steel were composed of Ni, followed in descending order by Al, Ca, Si, Mn, Cr, Cu, Mo, Mg, S, and Ti inclusions.

Among these inclusions, Ca inclusions have impurities remaining in the material or impurities caused by impurities during polishing. Ni and Cr inclusions are the strengthening elements of heavy-duty gear steels, so their effects on the bending fatigue performance of heavy-duty gears are not counted. Al, Mn, and Cu inclusions were refined in large quantities, while the Si inclusions did not change significantly, and the contents of other inclusions were too small. Among them, the most abundant were Al inclusions and the less obvious Si inclusions have attracted attention. The quantities of these two kinds of inclusions in the new type of steel is second only to that of Ni inclusions with metal reinforcement and Ca element inclusions included in itself and introduced from outside. In the process of steel smelting, there are more characteristic inclusions, and the following research mainly focuses on the effective analysis of these two kinds of inclusions, and according to the microfracture of the heavy-duty gear, the impacts of these two kinds of inclusions on the bending fatigue performance of a heavy-duty gear is analyzed. 

**Figure 6:**
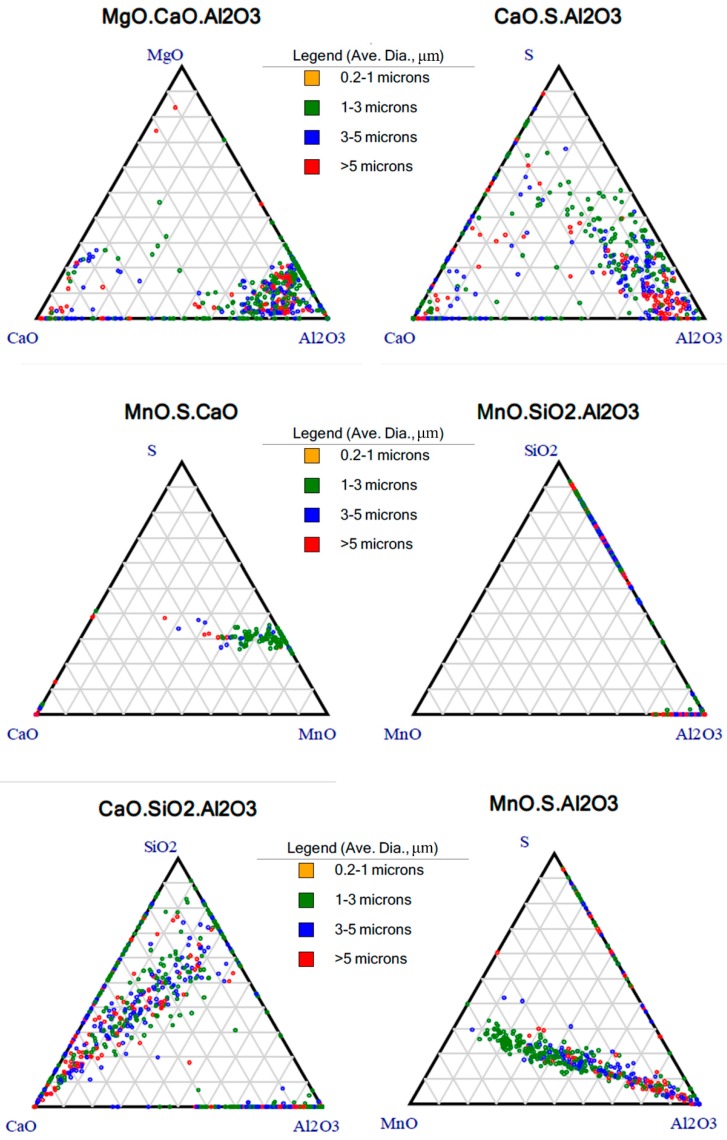
Ternary phase composition of the inclusions of 20Cr2Ni4 steel.

An Aspex automatic scanning electron microscope was used to count the inclusions in the samples (The test results are shown in Figure 6). The two types of steel inclusions detected were the same: Mainly MgO–CaO–Al_2_O_3_ and SiO_3_–CaO–Al_2_O_3_. The inclusions of this kind have a strong relationship with the conditions of steel in the furnace. By comparing the size and area of the inclusions, it can be found that a many large inclusions still exist in the ternary phase diagram distribution of steel inclusions of the old materials. These large inclusions are mostly MgO–CaO–Al_2_O_3_, CaO–S–Al_2_O_3_ and SiO_3_–CaO–Al_2_O_3_, and Al_2_O_3_ accounts for a large proportion of these inclusions. Al_2_O_3_ containing Al inclusions is the most common inclusion in real steel. There are various forms of inclusions in steel. Among them, the most important inclusions are calcium aluminate and Al_2_O_3_. Therefore, in some steel manufacturing processes, it is necessary to control the inclusion of calcium plagioclase, phosphorus quartz, and pseudowollastonite in the CaO–SiO_2_–Al_2_O_3_ series and the plastic inclusion in the adjacent low melting point zone. Al_2_O_3_ inclusions mostly come from inclusions left in the matrix before the Al_2_O_3_ particles separated from the molten steel are released after aluminum deoxidization is performed in the steel smelting process. Due to its strong oxyphilic ability, aluminum is a highly efficient deoxidizer, and the residual Al_2_O_3_ inclusions have difficulty forming plastic inclusions with low melting points. In the subsequent rolling process, the flow trend is relatively slow, resulting in an uneven structure, which has a great impact on the properties of steel. The Al_2_O_3_ inclusions will form the spinel structure of MgO·Al_2_O_3_ when MgO-based refractory is added in the smelting process. The inclusion of the spinel structure is a very harmful impurity to steel. In current research papers, most of the spinel structure MgO·Al_2_O_3_ is modified into liquid calcium aluminate.

**Figure 7:**
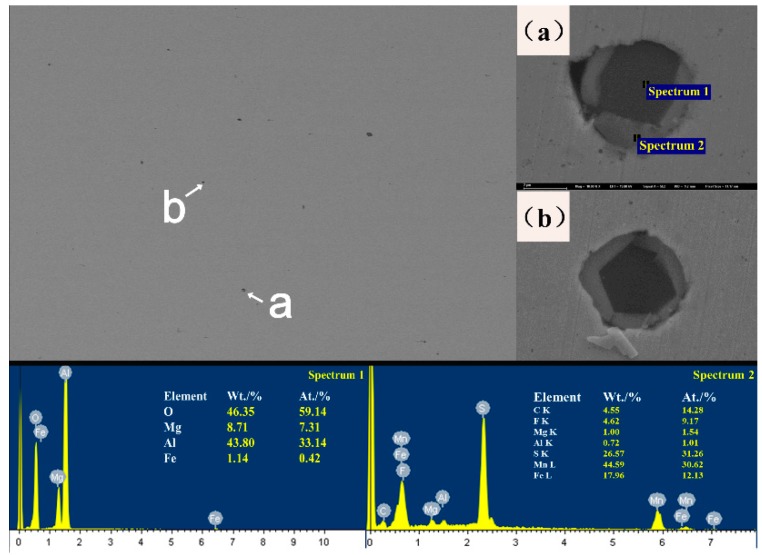
Scanning morphology and energy-dispersive X-ray spectroscopy (EDS) of material inclusions. (**a**): magnified image of the inclusions at a (**b**): magnified image of the inclusions at b.

Then, the status of the inclusions in the steel was analyzed, and the typical scanning morphology is shown in Figure 7. After statistical analysis, the average size of the inclusions is 5–10 μm, which are mostly circular. In some inclusions, there is only one phase, and some inclusions are a mixture of two phases. Two-phase mixed inclusions were analyzed by their energy spectra, as shown in Figure 7a,b. The black phase was mainly composed of the oxide of Al and Mg, while the white phase was the oxide of S and Mn. Al and Mn are caused by adding Al and Mn as the deoxidizer to produce Al_2_O_3_ in the process of the steel converter. In the subsequent process, after the transformation of the inclusions in the MgO·Al_2_O_3_, the outer surface is covered with black oxides of S that have not yet been removed. After feeding the Al line in steel, the spheroidization degrees of the inclusions are improved, but the Al_2_O_3_ and spinel structure of MgO·Al_2_O_3_ are still very hard and have very high melting points. Overall, the Al_2_O_3_ and spinel structure of MgO·Al_2_O_3_ will reduce the fatigue resistance of steel.

### 3.2. Fatigue Behavior Analysis

**Figure 8:**
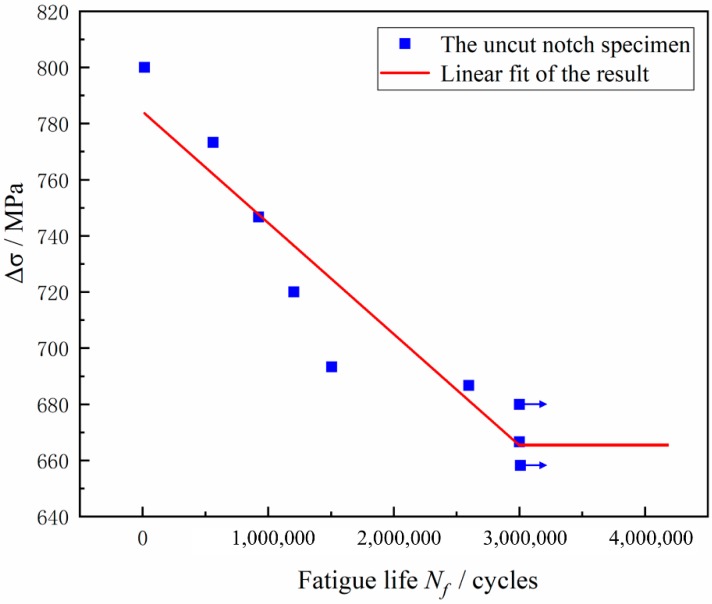
S-N curves of the 20Cr2Ni4 steel samples.

The S-N curve of a metal component is used to show the relationship between the stress and the fatigue life experienced by a component during testing and is the basic tool for analyzing the carrying capacity of a component. First, the fatigue life curve of the original three-point bending fatigue specimen was generated, and the test data points measured at each stress level were plotted as scatter plots. To ensure that all data points were concentrated in the same area, the horizontal and vertical axes are selected in decimal scale, and the natural logarithmic coordinate system is not used, as shown in Figure 8. Because the data points of flexural fatigue life are obviously dispersed, the intrinsic characteristics of the research objects cannot be directly revealed, and the test data need to be processed and analyzed. Therefore, the least-square fitting was performed for the logarithmic data points, and the fitting curve equation was selected as the Basquin Equation:

(1) $$\sigma_{max}^{m}\times N=C$$

In the expression, C and m are the desired parameters, N is the fatigue life value actually measured in the test, and$\;\sigma_{max}^{m}$ is the maximum loading value of steel during the test. The S-N curve with a survival rate of 50% was obtained by the least-square regression calculation. The expression for these two curves is as follows:

(2) $$\Delta \sigma =784.302-0.00003960\;N_{f}$$

Δσ is the loading value of steel during the experiment, and $N_{f}$ is the fatigue life In practical applications of S-N curves, it is necessary to find not only the slant equation of a finite domain but also the value of the turning point of the infinite life of the bending fatigue limit σ_Flim_ (the number of cycles is 3 × 10^6^). The design of the gear structure and the allowable stress determination provide a reference and basis. Therefore, in this project, 3 × 10^6^ cycles is used as the inflection point of the infinite life zone of the S-N curve, and the calculation is σ_Flim_ = 665.502 MPa.

**Figure 9:**
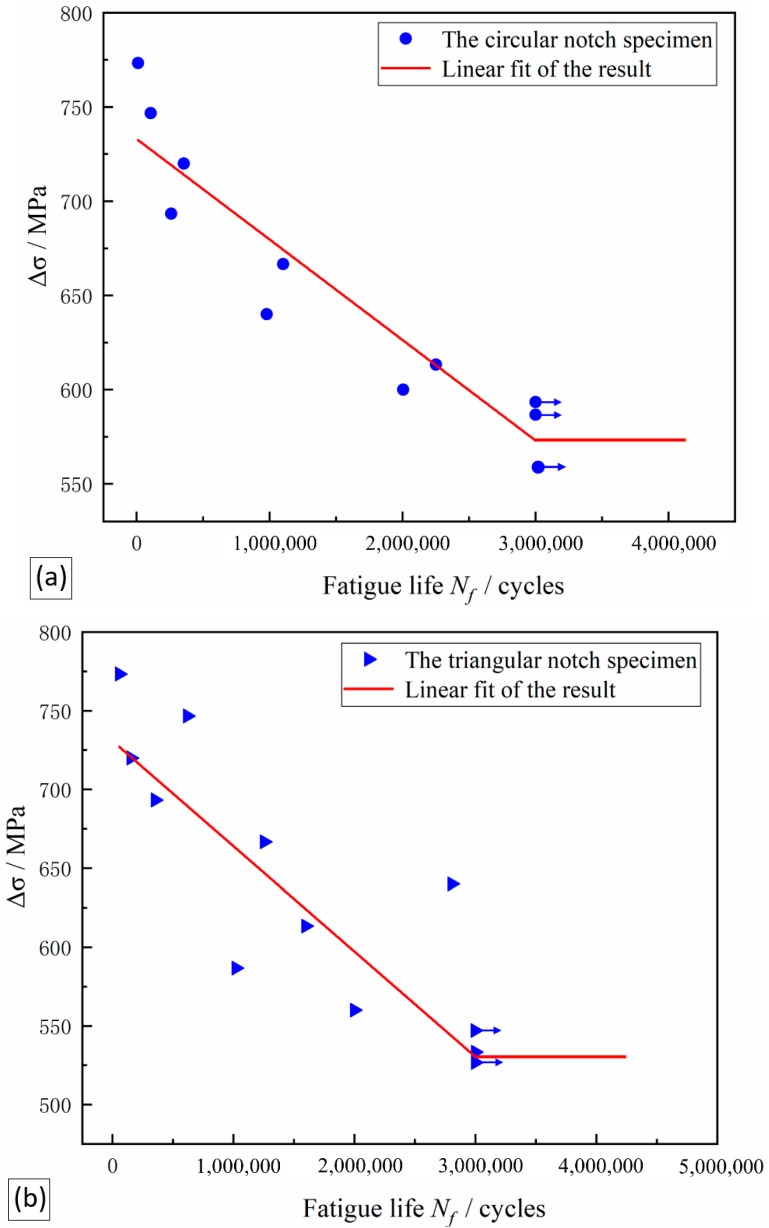
S-N curves of the notched specimens: (**a**) circular notched specimens and (**b**) triangular notched specimens.

The above method is used to calculate the data of circular and triangular notched specimens The S-N curve drawn by the same calculation method as above. The test results of circular notched specimens are shown in Figure 9a, and the triangular notched specimens are shown in Figure 9b. The expressions of these two curves are as follows:

(3) $$\Delta \sigma =773.063-0.00005334\;N_{f}\times \mathrm{Round}$$

(4) $$\Delta \sigma =730.828-0.00006679\;N_{f}\times \mathrm{Triangle}$$

Therefore, in this project, 3 × 10^6^ cycles is used as the inflection point of the infinite life zone of the S-N curve, and the fatigue limit of the circular blind hole sample is calculated as σ_1Flim_ = 573.028 MPa, and the fatigue limit of the triangular blind hole sample is σ_2Flim_ = 530.458 MPa.

### 3.3. Fractography Morphology

**Figure 10:**
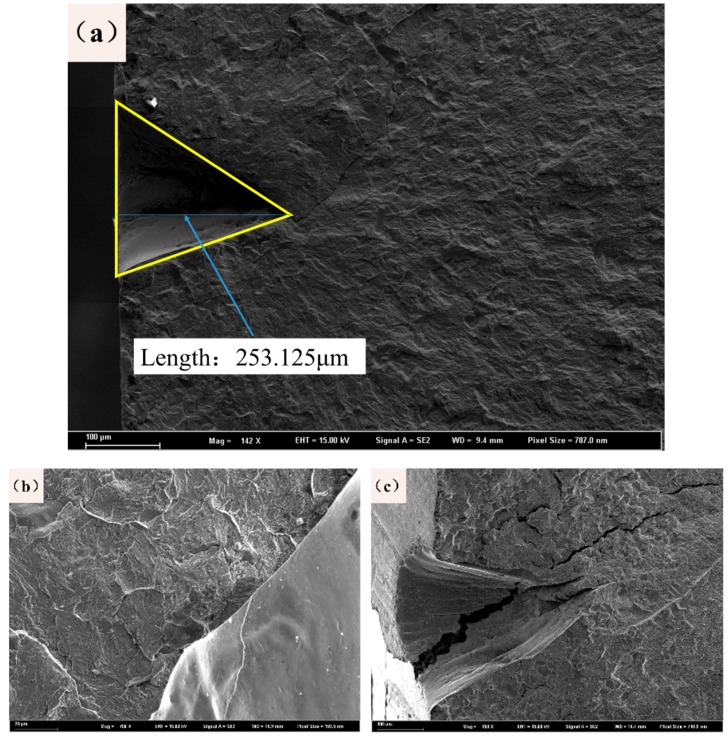
Fracture morphologies near the blind hole: (**a**) blind hole depth measurement graph, (**b**) normal sample morphology, and (**c**) stress concentration of large sample shape.

Figure 10 shows the fracture morphologies near the laser-prefabricated blind holes. First, it is confirmed that the technology is more suitable for improving bending fatigue performance than other incisions. Figure 10a is used to calculate the depths of the prefabricated defects, and the blind hole depth is 200–300 μm. Figure 10b shows an enlarged scanned image of a partially blind hole area. There is no obvious crack near the blind hole, the surface smoothness is good, and there is no rough structures or burrs. This preformed defect is a successful sample in this experiment. However, in other group experiments, there are also samples with poor smoothness, as shown in Figure 10c, which is related to the loading power, the loading cycle number, and the sample performance used in the laser etching. Second, in the subsequent fatigue process, the source of the crack is directly separated from the sample surface or directly disconnected from the defect, avoiding the expected fracture position. This result is mainly due to the small cracks around the prefabricated defects or the stress concentrations at the processing defects, which resulted in uneven loading of the samples during the experimental process.

**Figure 11:**
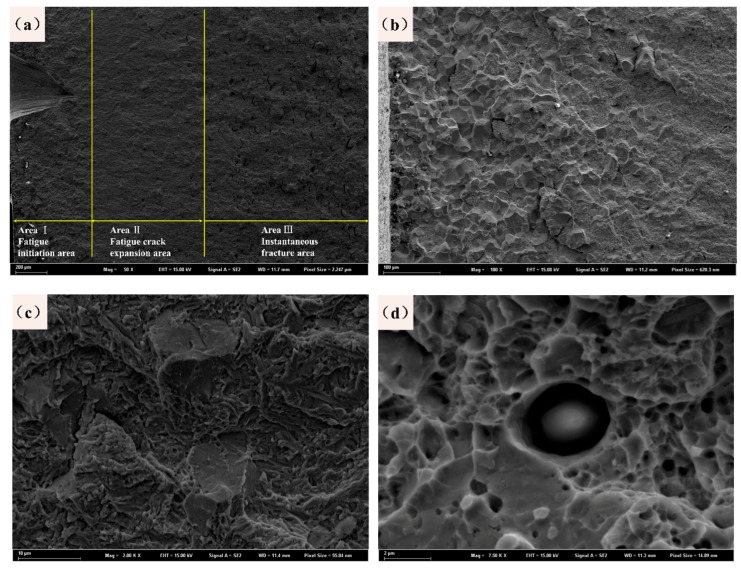
Fatigue fracture morphologies of the sample after three-point bending fractures: (**a**) overall morphology, (**b**) fatigue initiation area, (**c**) fatigue expansion area, and (**d**) instantaneous fracture area.

The fracture morphology of the sample was observed by SEM, as shown in Figure 11a. The fatigue fracture morphology can be clearly divided into three regions with different morphological features. The crack initiation area is a part of the interface to the prefabricated defect, which can be approximated as a fan-shaped pattern. Because the prefabricated notches provide a strong stress concentration, this region has become a typical fatigue source and is also the location of the fan handle. An enlarged morphology of this part is shown in Figure 11b. This area repeatedly opens and closes during the fatigue process, causing the fracture to grind on both sides. The appearance of the fracture surface is mostly a large-grained oceanic pattern. In the area after the crack initiation zone, the internal fracture morphology of the sample shows a distinct feature of fatigue crack propagation. From Figure 11c, the characteristics of quasi-cleavage fracture can be observed. The quasi-cleavage fracture extends along a certain crystalline surface, and its plastic deformation is greater than that of a cleavage fracture and less than that of a ductile fracture. Therefore, the quasi-cleavage fracture belongs to a brittle transgranular fracture. Quasi-cleaved facets can be found in this area with small secondary cracks and fatigue striations. In Figure 11d, bright white small particles can be found, where the alumina phase encloses a ring of carbides and the inclusions do not produce the same strain as the matrix when plastically fractured.

## 4. Discussion

### 4.1. Mechanism of Laser Etching to Predict Fatigue Performance

**Figure 12:**
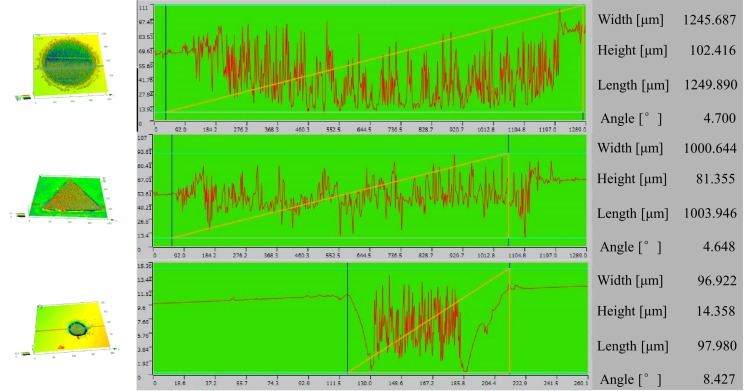
Three-dimensional topography of a blind hole.

From the test method introduced in Section 2.2, it can be seen that the innovation point of this research is to use the marking positioning technology of a microscope to realize the function of processing arbitrary patterns at arbitrary positions of the sample. The method first uses a microscope to observe the position of the inclusions on the sample and makes appropriate marks in the vicinity of the inclusions. By using laser etching equipment to make a blind hole at the mark, the blind hole covers the indentation from the microhardness testing to eliminate the possibility of hardness indentation acting as a new crack source. This method can observe the state of the inclusions in the fatigue stage after prefabrication with different stress concentrations. The results of the blind holes were observed by a three-dimensional profiler (as shown in Figure 12). Due to the experimental design, the resolution at the over depth position is too low and the data accuracy is poor. Therefore, all blind holes are guaranteed to be in a deep position by means of processing the same cycle. After comparing the depths, actual diameters, and indentation coverage of the blind holes under different processing parameters, the following experimental rules are summarized:

1. The microhardness tester results can be seen by the naked eye with three regular indentations.
2. The observation of inclusions must be ultimately supplemented by energy spectra for final confirmation, which requires multiple experiments to ensure the effects.
3. The parameters of the laser etching equipment are set in the diameter of 0.2 mm–1mm to ensure full coverage of the indentation, and the power of 50 W and 200 cycles can reach the desired depth.

**Figure 13:**
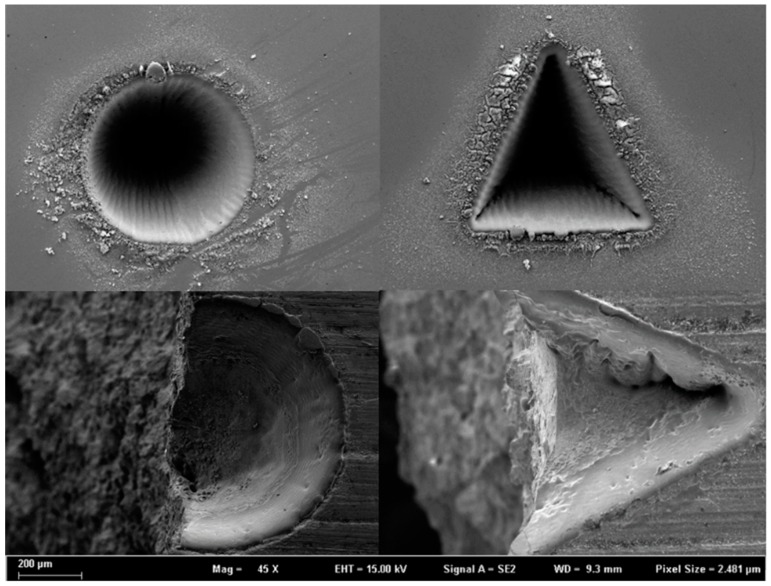
Morphologies of the blind holes before and after fracture.

It can be seen from the microscopic fracture morphology in Section 3.3 that the artificial damage caused by the laser-prefabricated defect method is small. This defect fabrication method can make the minimum diameter of the defect reach 10 μm so that there will be a greater chance of avoiding the key observation position of the sample and protecting the texture of the sample. Next, through SEM observations, the morphologies of circular and triangular blind holes made by the laser etching apparatus and the corresponding fractured topography are shown in Figure 13, and it can be seen that there are melted metal droplets attached to the surface layer around the small holes. However, there are no obvious cracks inside the blind holes. These metal droplets can be eliminated with the subsequent polishing process to reduce the possibility of melted droplets becoming crack sources. The lower part of Figure 13 shows the morphology of the corresponding specimen after fracture. It is obvious that the initial position of the fracture is in the stress concentration of the blind hole, and it is convenient to study the effects of the stress concentration of different precast shapes on the fatigue property of the matrix.

The experimental method can locate the influence of different stress concentrations on the inclusion in gear steel. The experimental method uses a specific distance around the inclusion to produce a certain stress amplitude to analyze the stresses of inclusions rather than simple qualitative research to verify the finite element calculation software or the actual operating conditions of the experimental results. Moreover, according to the fracture surface morphology, it can also be seen that the method does not produce obvious cracks, and the fatigue fracture is caused as a result of local stress concentration; therefore, the experimental method gives a complete analysis of the fatigue failure mechanism.

### 4.2. Influence Mechanisms of the Inclusions and Prefabricated Stress on the Fatigue Properties

SEM was used to characterize the fracture surfaces of selected test specimens to find inclusions, voids or any material defects at the crack initiation sites. Most of the cracks in the fracture surface will appear with inclusions. After statistical analysis, in this experiment, the size of inclusions in the steel is between 5 and 10 μm, and the chemical composition is mainly aluminum, sulfur, silicon, and some magnesium, manganese, and molybdenum.

**Figure 14:**
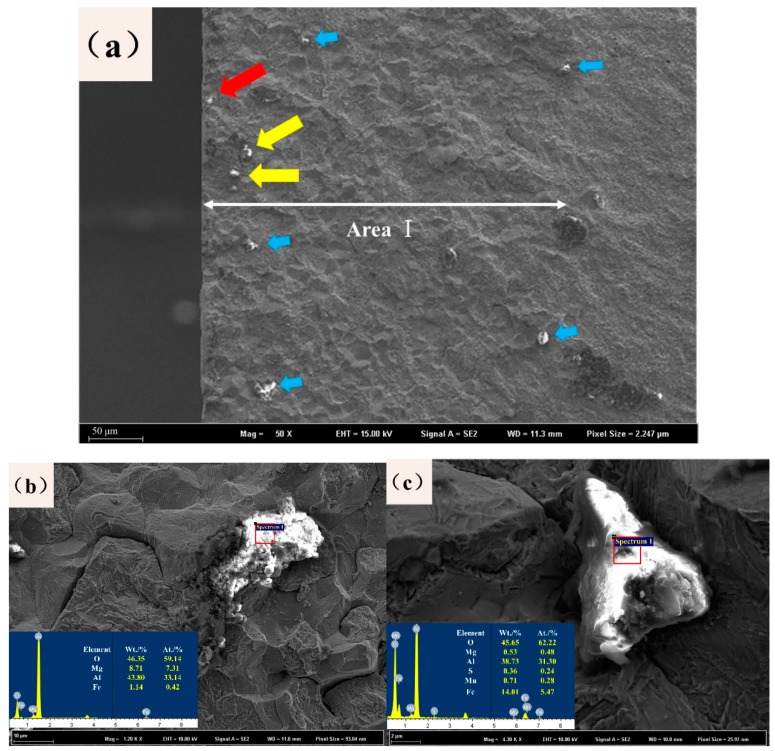
Fatigue fracture morphology of the sample after three-point bending fractures: (**a**) appearance of area I, (**b**) cracks in the inclusions, and (**c**) crack passing through the inclusions.

The inclusions are involved in each fatigue process, they are closely bound with the matrix, but their own elastic moduli are different from that of the matrix. Plastic deformation occurs during the service of steel, and the shape variables of the inclusions are inconsistent with that of the matrix, which results in the failure phenomenon. Due to severe stress concentrations in the near-surface inclusions, their failure behaviors will be given priority, and the failure behavior of the fatigue region can be observed from Figure 14a. The red arrow marks the starting point of the visible crack, which extends to the second crack initiation point where the yellow arrow is located; the crack then extends to the next area shown by the blue arrow. Moreover, once the expansion of the crack occurs in the blue arrow inclusions in the subsequent process, it will change direction at a time and cause more serious damage effects [20]. Enlarged typical position morphologies of the compared gap are shown in Figure 14b,c. Figure 14b shows that the cracks produced by the inclusion cause damage to the substrate according to the state and the extension of the inclusions. Figure 14c shows that the cracks in the expansion process encounter obstructions from the inclusion and change the crack propagation direction. Therefore, to improve the fatigue performance of metal components, it is necessary to start by reducing the harmful effects of inclusions. 

The improvement in bending fatigue performance is mainly due to the control of the harmful effects of inclusions. The inclusions mainly consist of brittle Al_2_O_3_ inclusions, which seriously damage the continuity of the steel matrix and have a great influence on the brittleness and fatigue properties of the gear steel. Sulfide is also a harmful element in steel, which makes the steel hot and brittle, reduces the ductility, toughness and corrosion resistance of the steel, and the welding performance of the steel is also unfavorable. However, steel with a high sulfur content can form more MnS, which can improve the cutting performance of steel. Nevertheless, the effects of sulfides on steel tend to be detrimental. Therefore, sulfides are also harmful inclusions that need to be controlled in the steel. The reduction in the number or size of inclusions can obviously improve the bending fatigue performance of steel. In addition, excessive stress concentrations near inclusions can also cause fatigue failures. Thus, we need to further improve the bending fatigue performance of the heavy-duty gear steel from the perspective of stress concentration, which requires the prestressing method in the vicinity of inclusions designed in this paper. By comparing the S-N curves of the triangular notched samples and the circular notched samples, the fatigue limits of these samples can be calculated as 530.458 MPa and 573.028 MPa, respectively. This difference in fatigue life is caused by different stress concentrations in the samples: The circular notch is more uniform than the triangular notch, and thus the circular notch can pass the load to the surrounding material. As such, the circular notch can effectively share the inclusion bearing pressure, which gives the circular notched samples better fatigue performance than the triangular notched samples. Therefore, the laser etching machine can be used to make laser blind holes with a minimum diameter of 10 nm. It is convenient to design blind holes that can provide different stress concentrations and accurately distribute the load on heavy-duty gear inclusions. The parts can effectively share the stress concentration on the inclusions, thereby improving the bending fatigue performance of heavy-duty gears.

### 4.3. Fatigue Strength Prediction of 20Cr2Ni4 Steel Notch Shape and Inclusion Size

The fatigue strength of steel has the property of highly variable random failures. This property is related to several factors, such as the metallurgical quality of the steel itself, the damage during processing, and the error caused by fatigue testing. Therefore, the research on fatigue is mostly focused on the accuracy of predicting the fatigue strength because the fatigue strengths, fatigue lives, and fatigue limits of materials are mostly predicted. Compared with the fatigue life dispersion, there are a greater number of influencing factors for fatigue strength. The fatigue strength fitting of the 20Cr2Ni4 material is performed. It is worth mentioning that the empirical formula of the fatigue strength of the inclusion size proposed by Murakami is studied. According to the Paris formula, as a basic theory of research, the stress concentration around the inclusion is added to correct the formula proposed by Murakami.

First, the Murakami and Endo [18] model is used to study the inclusion area and matrix hardness value, and the size of the inclusions is quantified so that the inclusions have an area size criterion in the steel; then a substantial amount of experimental verification is performed. The empirical formula for the Murakami and Endo model and some similar mathematical models is as follows:

(5) $$\sigma_{w}=\frac{1.43\left(H_{V}+120\right)}{{\left(\sqrt{area}\right)}^{1/6}}$$

In this paper, based on Equation (4), according to the Paris formula (As shown in Equation (5)), the stress correction factor k is added, the physical meaning of k is the radius of curvature of the prefabricated blind hole under the determined circumcircle diameter. The reference of this factor is related to the fracture factor ΔK. The fracture factor ∆K is mainly related to the stress experienced by the material fatigue process and the defects in the material, so this factor is also added to the model. Correction of the fracture factor ∆K can better improve the accuracy of the mathematical model.

(6) $$\mathrm{da}/\mathrm{dN}=C{\left(\Delta K\right)}^{m}$$

In the formula, da/dN is the crack growth rate, and c and m are the constants of the material, ΔK is mainly related to the fracture morphology of the prefabricated steel. According to the data values given in Section 3.2, ∆K is considered to be related only to the shape and size of the prefabricated gap when other variables are controllable. In mathematics, the curvature k can be introduced to characterize the size of the geometry and the sharpness of the figure.

**Figure 15:**
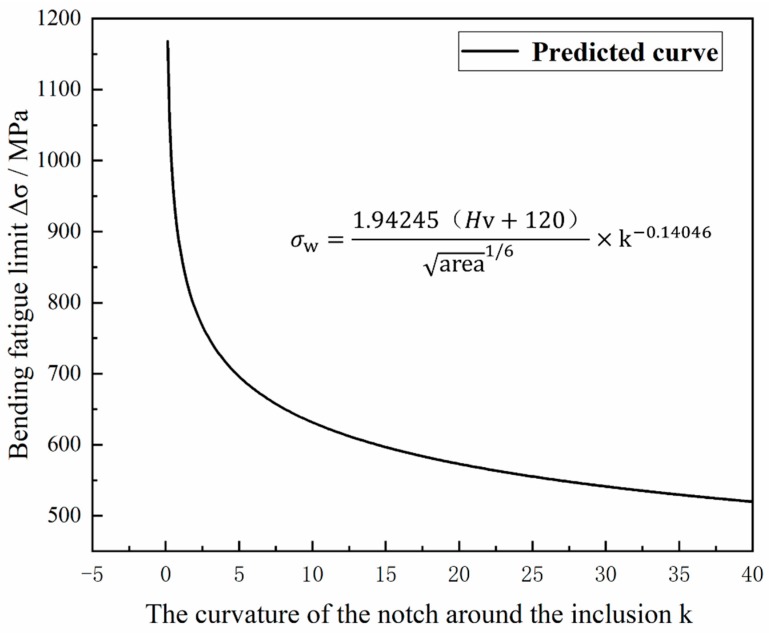
New model fitting curve.

In summary, according to the integration of Equations (4) and (5), the modified parameters C and m are introduced, and a mathematical model of the fatigue limit $\sigma_{w}$, the average size of the inclusions $\sqrt{area}$, the material hardness value $H_{V}$, and the radius of curvature r of the preformed gap is proposed:

(7) $$\sigma_{w}=\frac{C\left(H_{V}+120\right)}{{\left(\sqrt{area}\right)}^{\frac{1}{6}}}\times {\left(k\right)}^{m}$$

where $\sqrt{area}$ is the square root of the average size of the inclusions in the steel, which was determined in Section 3.1 as 12.34 μ; Hv is the hardness of the steel, which was determined in Section 2.1 as 434 HB; k is the stress intensity factor, which is expressed in this experiment as the influence of the curvature of the defect near the inclusion on the fatigue performance; and $\sigma_{w}$ is the bending fatigue limit of the steel. Then, data fitting is performed to obtain the values of the modified parameters C and m. The two sets of test data in Section 3.2 can be used to determine that C = 1.94245 and m = −0.14046. The final expression is shown in Equation (6), and the fitting curve is shown in Figure 15:

(8) $$\sigma_{w}=\frac{1.94245\left(H_{V}+120\right)}{{\left(\sqrt{area}\right)}^{\frac{1}{6}}}\times {\left(k\right)}^{-0.14046}$$

**Figure 16:**
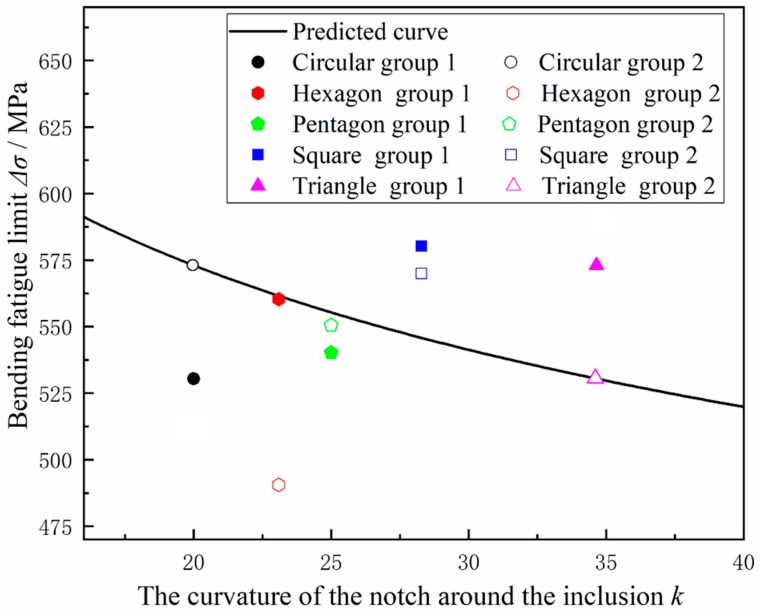
Comparison of the predictions by Equation (6) with the actual experimental fatigue lives.

The scope of the model is mainly applied to the fatigue testing of heavy-duty gear steel. The input parameters are the hardness value of the steel, the square root of the average size of the inclusions in the steel, the curvature of the defect near the steel inclusions. Finally, the fatigue limit can be calculated. The model creates a local stress concentration structure of the material by forming defects around the inclusions to create a localized stress concentration structure of the material to quantitatively indicate the local stress concentration and the influence of inclusions on the fatigue properties of the steel. Subsequently, the experiment was performed to verify the accuracy of the model. According to the above test method in Section 2.2, square notches, pentagonal notches and hexagonal notches under the same circumcircle diameter with the Section 2.2 triangular and circular cuts near the inclusions are treated, and the fatigue limit was tested according to the fatigue test procedure in Section 2.2. The prediction curve was introduced into the prediction curve of Figure 15 to form Figure 16. The point is basically near the straight line, and the calculation results basically conform to the prediction relationship, but since the parameter of fatigue limit itself is a highly random variable, the regression analysis results still cannot provide significant proof.

## 5. Conclusions

In this paper, the three-point bending fatigue behavior of heavy-duty 20Cr2Ni4 gear steel with different inclusion contents is studied. In this study, a method of adding positioning defects was introduced to evaluate the fatigue properties of steel inclusions. From the experimental results and analysis, the following conclusions can be drawn:

1. The method of laser positioning and adding defects can improve the stress concentration of prefabricated inclusions. Compared with wire cutting technology and FIB, this method reduces the damage and makes the sample more suitable for fatigue research. Moreover, this method can provide experimental samples for finite element software to predict the influence of inclusion type, force size and spatial distribution on the fatigue life of a material.

2. Under standard experimental conditions, the fatigue limit of the heavy-duty 20Cr2Ni4A gear steel is 665.502 MPa. Fracture analysis reveals that the presence of inclusions is the main factor leading to fatigue fracture. To study the effect of stress concentration on the bending fatigue performance, an Aspex automatic scanning electron microscope was used to analyze and count the inclusions in the steel samples. The inclusions in heavy-duty gear steel were MgO-CaO-Al_2_O_3_ and SiO3-CaO-Al_2_O_3_. These two types of inclusions are well distributed, and the distribution is relatively uniform; the inclusion area is between 1 and 5 μm.

3. The fatigue limits of the triangular notched specimen and the circular notched specimen measured under the same experimental conditions were determined to be 530.458 MPa and 573.028 MPa, respectively. After the fracture analysis, it was found that the presence of inclusions and local stress concentration are the main causes of fatigue fracture. According to the hardness of the steel, the value of hardness, the square root of the average size of the inclusions in the steel, and the curvature k of the defect near the steel inclusions (among other parameters) are modelled by the bending fatigue limit fitting, and a new mathematical model is given to predict the overload. The fatigue limit of gear steel is expressed as Equation (7). The principle of the model is to provide a new method for improving the bending fatigue life. By designing blind holes of different shapes, the positioning technology is used to prefabricate holes in the vicinity of a large concentration of inclusions, thereby affecting the surrounding stress distribution of the steel and finally affecting the concentration of fatigue; this process was verified through experiments. The degree of model fitting is good to improve the stress in a specific area, establish an effective load-bearing structure, and improve the fatigue performance of the gear steel.

## Figures and Tables

**Table 1:** 20Cr2Ni4 steel element content components.

Process Standard	Alloy Element (wt%)							
	C	Cr	Ni	Mn	Si	Al	S	O
National standard	0.17 ~ 0.23	1.25 ~ 1.65	3.25 ~ 3.65	0.30 ~ 0.60	0.24	≤0.03	≤0.03	≤0.0025

**Table 2:** Mechanical properties of 20Cr2Ni4.

Steel Grade	Tensile Strength (MPa)	Elastic Modulus (MPa)	Yield Strength (MPa)	Section Elongation (%)	Section Shrinkage (%)	Hardness (HB)
20Cr2Ni4	1483	211	1292	13	57	434

**Table 3:** Temperature of the heat treatment process for gear steel.

Steel	Heat Treatment Process				
20Cr2Ni4	Normalizing Temperature /°C	Carburizing temperature /°C	High-temperature Tempering/°C	Quenching Temperature /°C	Low-temperature Tempering/°C
	950	920	640	800	150
